# Manipulation of BDNF Signaling Modifies the Experience-Dependent Plasticity Induced by Pure Tone Exposure during the Critical Period in the Primary Auditory Cortex

**DOI:** 10.1371/journal.pone.0064208

**Published:** 2013-05-21

**Authors:** Renata Anomal, Etienne de Villers-Sidani, Michael M. Merzenich, Rogerio Panizzutti

**Affiliations:** 1 W.M. Keck Foundation Center for Integrative Neurosciences, University of California San Francisco, San Francisco, California, United States of America; 2 Program for Basic-Clinical Neuroscience, Institute of Biomedical Sciences, Federal University of Rio de Janeiro, Rio de Janeiro, Rio de Janeiro, Brazil; University of South Florida, United States of America

## Abstract

Sensory experience powerfully shapes cortical sensory representations during an early developmental “critical period” of plasticity. In the rat primary auditory cortex (A1), the experience-dependent plasticity is exemplified by significant, long-lasting distortions in frequency representation after mere exposure to repetitive frequencies during the second week of life. In the visual system, the normal unfolding of critical period plasticity is strongly dependent on the elaboration of brain-derived neurotrophic factor (BDNF), which promotes the establishment of inhibition. Here, we tested the hypothesis that BDNF signaling plays a role in the experience-dependent plasticity induced by pure tone exposure during the critical period in the primary auditory cortex. Elvax resin implants filled with either a blocking antibody against BDNF or the BDNF protein were placed on the A1 of rat pups throughout the critical period window. These pups were then exposed to 7 kHz pure tone for 7 consecutive days and their frequency representations were mapped. BDNF blockade completely prevented the shaping of cortical tuning by experience and resulted in poor overall frequency tuning in A1. By contrast, BDNF infusion on the developing A1 amplified the effect of 7 kHz tone exposure compared to control. These results indicate that BDNF signaling participates in the experience-dependent plasticity induced by pure tone exposure during the critical period in A1.

## Introduction

The critical period is an initial postnatal epoch of cortical development that is highly susceptible to the plasticity induced by environmental stimuli [1]. During the critical period, the electrical activity generated by sensory experiences modulates the organization of cortical maps through strong expansion or retraction of cortical and subcortical connections [2], [3]. The refinement of intracortical circuitry across this development period has impacts on sensory perception of adult life. An understanding of the mechanisms that regulate it is fundamental to understanding how disorders of perceptions are generated, and potentially, how they could be avoided or overcome.

The opening and the closure of the critical period vary in different sensory modalities [4] and as a function of neuronal properties [5]. The exposure of rats to pure tone during the critical period augments the representation of that stimulus in the primary auditory cortex (A1) [6], [7]. Using pure-tone exposures, de Villers-Sidani et al. (2007) [8] recorded persistent alterations in sound representations in A1 only if that exposure occurred during a brief period extending from postnatal day 11 (P11) to P13, defining the critical period for spectral tuning in this region. Further studies indicated that critical period closure in A1 was locally controlled [9].

Studies have showed that neurotrophins control the onset and closure of critical period as well as the magnitude of experience-dependent plasticity in the primary visual cortex (V1). The blockade of the brain-derived neurotrophin (BDNF), in an early postnatal epoch blocks the development of ocular dominance columns in V1 [10], [11]. Moreover, precocious expression of BDNF in transgenic mice accelerates the maturation of visual acuity [12]. These effects of BDNF in V1 parallel and arguably participate in the maturation of cortical inhibitory circuitry. In V1 development in mice, exposure of visual cortex to BDNF accelerates emergent GABAergic inhibition, which results in an earlier critical period closure [1], [12].

We recently observed that the restoration of the critical period of plasticity in the adult A1 by chronic exposure to acoustic noise was followed by reduced cortical expression of BDNF [13]. However, BDNF has not been shown to play a role in experience-dependent plasticity in A1 during the critical period. The present study was designed to determine whether BDNF modulates experience-dependent plasticity induced by pure tone exposure across the critical period for spectral tuning in A1. Elvax resin filled with either a blocking antibody against BDNF or BDNF were implanted over A1 just before critical period opening. Rats were then exposed to a continuous pure tone for a 7-day epoch extending across the critical period window. At the end of that exposure period, we mapped the electrophysiological receptive field to determine, by reference to control age-matched rats, whether or not BDNF or BDNF blocking altered stimulus-induced critical-period changes in A1.

## Experimental Procedures

All procedures were approved by the animal care committee of the University of California in San Francisco. We studied 17 Sprague Dawley rats. In all animals, an Elvax resin implant was mounted over the auditory cortex in the right hemisphere at P9. The implant resin was loaded with either an antibody to BDNF (Millipore, Billerica, MA) (1 mg/ml), with the BDNF protein (Sigma-Aldrich, St. Louis, MO) (0.1 mg/ml), or with vehicle.

Elvax beads (Du Pont, Wilmington, DE) were washed in distilled water followed by 95% and 100% ethanol. Washed Elvax was dissolved in 10% methylene chloride and 2% fast green was added. The prepared Elvax was frozen and kept at −70°C for 1 hour and at −20°C overnight, then cut into 60 µm thick sections on a cryostat and stored until use.

To implant the resin, rat pups were anesthetized with isofluorane and a small craniotomy was performed to expose the primary auditory cortex under sterile surgical conditions. An incision was then made in the dura and the resin was placed subdurally on the cortex and centered over A1. The skull fenestration was then covered with a dental acrylic cap affixed with dental cement, and the temporal muscle and skin sutured back in place.

### Sound exposure of rat pups

All implanted pups and their mothers were placed in a sound-shielded test chamber from P9 to P16. Rats were continuously exposed over this 7 day period to trains of 5 successive 25-ms-long 7 kHz tone pips delivered at a sound level of 70 dB SPL with 500-ms quiet intervals between the tone pip trains. The weights of all rats were monitored continuously; no weight losses compared with naive rats were recorded, indicating normal lactation. The activities during wakefulness and the sleep behavior of the rats revealed no abnormality.

### Mapping the auditory cortex

Two days after the end of the pure tone exposure, the auditory cortex of rat pups was mapped as previously described [8]. Animals were anesthetized with Nembutal (60 mg/kg) and administered anti-inflammatory dexamethasone (0.2 mg/Kg) and atropine (0.02 mg/Kg) to avoid mucus secretion. Body temperature was maintained with a heating pad, and heart rate continuously monitored. The cisterna magnum was drained of cerebrospinal fluid to minimize cerebral edema. The skull was secured in a head holder, leaving the ears unobstructed. The right temporalis muscle was reflected, auditory cortex was exposed, and the dura mater resected to broadly exposure A1.

Electrophysiological recording was performed with pair low-impedance tungsten microelectrodes (1–2 M at 1 kHz; FHC Inc., Bowdoinham, ME). Microelectrodes were lowered perpendicular to the cortical surface to depths of 470 to 600 µm (layers 4 and 5). Each penetration site was marked at a high-resolution image of the brain surface. The average nearest-neighbor recording site distance was approximately 250 to 500 µm. For each recording site, the contralateral cochlea was stimulated by acoustic stimuli generated by TDT System III (Tucker-Davis Technologies, Inc., Alachua, FL), delivered to the cochlea through a calibrated earphone (STAX 54) sealed within the ear canal. Pure tones were presented at 50 frequencies (1–30 kHz, 0.1-octave increments, 25-ms duration, 5-ms ramps) at eight sound intensities (0–70 dB SPL in 10-dB increments) at a rate of 2 stimuli per second.

The neural signal recorded was amplified (10.000X), filtered (0.3–3 kHz) and monitored online. We used a software package (SigGen and Brainware; Tucker-Davis Technology) to generate acoustic stimuli, monitor cortical response properties on-line and store data for off-line analysis. The evoked spikes of multiple neurons were collected at each site. Recording sites in A1 typically presented strong responses evoked by low intensity tones. Recording sites outside A1 were responsive only to higher intensity sounds, or were not reliably excited by tonal stimuli [14].

### Data analysis

We identified the characteristic frequency of cortical sites (the frequency at which neurons responded at lowest threshold), and defined the response bandwidths above threshold (BW20) for all sites. The BW20 was defined as the range of frequencies in octaves that activated neurons at a recording site at 20 dB above threshold. Single-peaked sites were identified by one apex and a well-defined “V-shaped” tuning curve. When tuning curves had broad tips (multi/flat-peaked sites), the median frequency at minimal intensity was chosen as characteristic frequency. Characteristic frequencies, thresholds and BW20s were determined in the Matlab environment (The MathWorks, Natick, MA).

The cortical maps were generated by Voronoi tessellation, a MatLab function. The center of each polygon corresponds to the site of one penetration. The A1 border was determined by the response characteristics of recorded neurons and by the topographic progressions of represented frequencies in the cortex. The boundaries of the primary auditory cortex were functionally determined using the following criteria: (1) primary auditory neurons generally have a continuous, single-peaked, V-shaped receptive field, and (2) CFs of the A1 neurons are tonotopically organized with high frequencies represented rostrally and low frequencies represented caudally [14].

### Quantitative immunoblotting

The right A1 was rapidly dissected, frozen in dry ice and stored until processing at −80°C. For quantitative immunoblotting analysis, synaptoneurosomes were prepared as described by Hollingsworth et al. (1985) [15]. Equal amounts of synaptoneurosomal proteins (7–10 µg) determined using the BCA assay (Pierce, Rockford, IL), were resolved in 4–15% polyacrilamide gels and transferred to PVDF membranes. Membranes were probed with primary antibodies, followed by appropriate secondary antibodies conjugated with infrared dyes (LI-COR Biosciences, Lincoln, NE). The primary antibodies used were anti-GABA_A_ β2/3 (1∶1000, Upstate, Dundee, UK), and anti-γ-tubulin (1∶1000, Sigma-Aldrich, St. Louis, MO). Immunoreactive bands were visualized and quantified using Odyssey Infrared Imaging System (LI-COR Biosciences, Lincoln, NE). The relative levels of GABA_A_ β2/3 were calculated as a ratio against γ-tubulin.

### Statistics

Data are presented as mean ± SEM. Statistical significance was analyzed using ANOVA Turkey's test.

## Results

A control group (N = 9, 484 A1 recorded sites) received Elvax implants filled with vehicle. An anti-BDNF group (N = 4, 361 A1 recorded sites) received implants filled with a blocking antibody against BDNF. A BDNF group received implants filled with the BDNF protein (N = 4, 265 A1 recorded sites). To identify the effects of BDNF on experience-dependent plasticity, rats were exposed to 7 kHz pure tones across the critical period window for spectral tuning (P9 to P16; see [8]). A1 mapping was conducted 2 days after tone exposure was terminated.

**Figure 1:**
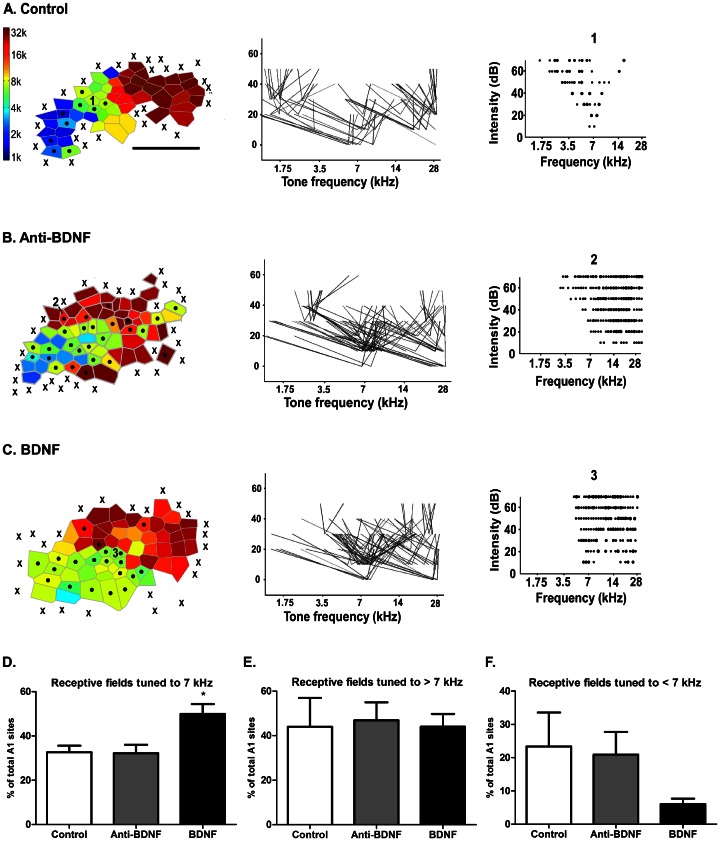
Manipulation of BDNF signaling during critical period affects the tonotopic organization, receptive fields and cortical representation of A1. (A, B, C) Tonotopic maps (left) of control, anti-BDNF, and BDNF groups; *x* = no tone-evoke responses; *circle inside polygon* = multi/flat-peaked sites. (Center) Distributions of tuning curves from illustrated map. The pair of joined lines represents the receptive field of each recorded site, and the apex indicates the threshold of activation of these sites. (Right) Representative receptive field from each displayed map (see number inside maps). (D) Receptive fields tuned to 7 kHz±0.3 octave were significantly increased in the BDNF group compared to control and anti-BDNF groups (F = 7.3, p<0.05, ANOVA). (E, F) The proportion of receptive fields tuned to frequencies higher than 7 kHz±0.3 octave (A) or lower than 7 kHz±0.3 octave (B) were not significantly different between groups. Values are means ± SEM. * p<0.05. Calibration bar = 1 mm.

In the control group, we observed the typical tonotopic orientation of A1, with sound frequencies from 1 to 32 kHz represented in bands following a caudal-to-rostral low-to-high-tone progression (Figure 1A) [8]. In controls, the percentage of A1 area containing receptive fields tuned to the exposure frequency (7 kHz±0.3 octave) was 32.7±2.9% after a week-long 7 kHz exposure protocol (Figure 1D). This is very similar to the percentage of 32.1±1.5% of A1 area tuned to 7 kHz reported by a previous study using the same exposure protocol [8]. The critical period for experience-dependent plasticity in A1 is linked to the emergence of fine-tuned cortical responses to auditory inputs, with most of the receptive fields becoming tuned across this period of maturation to a single frequency [8]. In Figure 1A, we also show a representative receptive field from the control group, illustrating the typical well-defined “V-shaped” of a single-peaked receptive field.

By contrast, A1 tonotopy was poorly organized in rats of the anti-BDNF group (Figure 1B). This degraded pattern of organization is similar to the immature state of A1 tonotopy after being driven back to an immature state by acoustic noise exposure [16]. In Figure 1B, we show an example of a flat-peaked receptive field receptive field, characteristic of the immature A1. Surprisingly, the percentage of site tuned to the exposure frequency (7 kHz±0.3 octave) in the anti-BDNF group (32.2±3.0%) was similar to the control group (32.7±3.8%) (Figure 1D).

In the BDNF group, the cortical representation of the exposure frequency 7 kHz in A1 was enlarged as compared to the control group (Figure 1C and 1D; BDNF = 50.0±4.4% *vs* Control = 32.66±2.99, F = 7.3, p<0.05). The percentage of A1 area containing neurons tuned to sound frequencies higher than 7 kHz±0.3 octave was similar in the three groups (Figure 1E; Control = 44.0±13.0%, anti-BDNF = 46.9±8.1%, BDNF = 44.0±5.7%), but the BDNF group showed a tendency (not statistically significant) to have reduced A1 area tuned to sound frequencies lower than 7 kHz±0.3 octave (Figure 1F; Control = 23.4±10.2%, anti-BDNF = 20.9±6.8%, BDNF = 6.0±1.6%).

**Figure 2:**
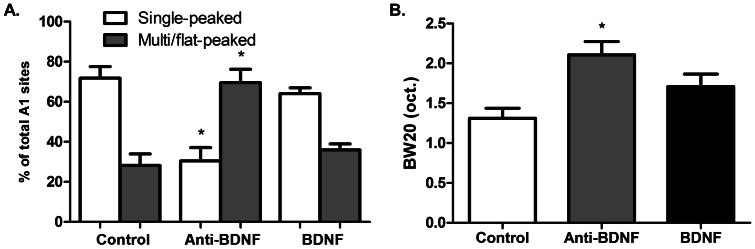
Manipulation of BDNF signaling affects spectral selectivity. (A) Percentage of single and multi/flat-peaked receptive fields for each study group. The percentage of single-peaked receptive fields was significantly reduced in the anti-BDNF group compared to controls (F = 13.6, p = 0.003, ANOVA). (B) Response bandwidths above threshold (BW20) were significantly augmented in the anti-BDNF group compared to controls (F = 7.3, p = 0.007, ANOVA). Values are means ± SEM. * p<0.05.

Neurons at the majority of sampled sites in control group (71.8±5.8%) had developed single-peaked receptive fields at the time of the electrophysiological mapping reconstructions (Figure 2A). The anti-BDNF group showed a significant increase in the proportion of sites with tuning curves with broad tips (multi/flat-peaked receptive field), again characteristic of the immature A1 (Figure 2A, anti-BDNF = 69.5±6.7% *vs* Control = 28.1±5.8%, F = 13.6, p = 0.003). We also defined spectral selectivity by calculating the BW20 (tuning bandwidth 20 dB above the unit's intensity threshold) (Figure 2B). Recorded neurons in the anti-BDNF group showed a significant increase in BW20 compared to the control group (Figure 2B). Recorded neurons in the anti-BDNF and BDNF groups did not differ in the thresholds or latencies of responses compared with one another, or to the neurons in the control group (data not shown).

**Figure 3:**
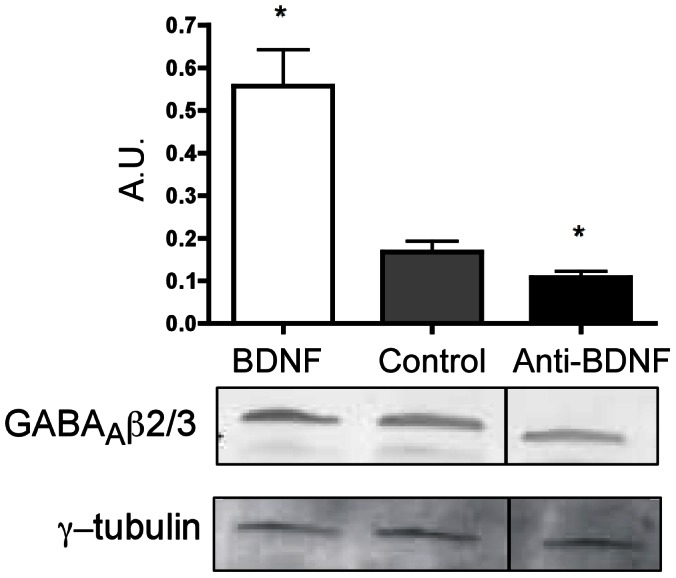
Manipulation of BDNF signaling affects the expression of GABA receptors in A1. Expression of GABA_A_ β2/3 subunits measured using quantitative immunoblotting in the different rat groups (3 animals per group). The insert shows a representative western blot. Values are means ± SEM. * significantly different than control rats at p<0.01.

The maturation of A1 during the critical period of plasticity is accompanied by the progressive maturation of inhibitory circuitry [9], [17]. This maturation is triggered by BDNF signaling, which induces the expression of GABA_A_ type of inhibitory receptors [18]. To determine if the manipulations that we used here had been effective in blocking or augmenting BDNF signaling, we determined the expression of the GABA_A_ β2/3 subunit in A1 (Figure 3). Confirming the biological action of our treatments, we observed reduced expression of GABA_A_ receptors in the anti-BDNF group compared to controls (Figure 3). Conversely and as expected, BDNF group rats showed increased expression of GABA_A_ receptors (Figure 3). We conclude that our treatment was effective in manipulating the biological effect of BDNF in A1. These changes in inhibitory GABA_A_ receptors plausibly contribute to the changes in A1 tuning recorded in this study.

## Discussion

Our results highlighted three important aspects of BDNF signaling in the experience-dependent plasticity induced by pure-tone exposure during the critical period for spectral tuning of rat A1. First, we showed that blocking BDNF signaling during the experience-dependent plasticity in the critical period for spectral tuning distorts the normally smooth A1 tonotopic axis. Second, infusion of BDNF in the A1 increased the representation of the exposed tone. Finally, we observed that our manipulations of cortical BDNF during the critical period were mirrored by alterations in the expression of GABA_A_ receptor subunits.

Taken together, these results indicate that the experience-dependent plasticity in A1 during the critical period for spectral tuning is affected by BDNF signaling. The blockage of endogenous BDNF in A1 during the critical period reduced the selectivity of neurons to tone frequencies; most of A1's receptive fields remained broadly tuned (multi/flat peaked with larger BW20s). It also affected the normal progressive development of the topographic order of A1. The blocking of BDNF signaling in A1 possibly affected the development of inhibitory circuitry known to mature across the critical period epoch. That maturation is known to play a role in the evolution of selective tuning for neuronal receptive fields in A1, as in other primary cortical areas [19], [20], [21]. Broadly tuned neurons and degraded tonotopy similar to that resulting from blocking BDNF has also been seen in rats that were continuously reared across the critical period and beyond in a continuous, moderate white noise environment [7]. As in the present study, that noise blocked A1 maturation and GABA_A_ β3 subunit expression [22], [23]. Interestingly and surprisingly the anti-BDNF group showed a large percentage of the A1 area tuned to the 7 kHz exposure frequency, similar to control exposed rats. This suggest that the anti-BDNF antibody was able to decrease the development of sharp selectivity of neurons to tone frequencies but it did not affect the definition of the best frequency of the neuronal response.

In the visual cortex BDNF regulates the maturation of inhibition and the critical period of plasticity [12]. Moreover, BDNF overexpression in V1 induces a precocious critical period, rescues the effect of dark rearing, and is sufficient for the development of aspects of the visual cortex in the absence of visual experience [24], [25]. We reported in Zhou et al. (2011) [13] that BDNF levels in the auditory cortex are decreased in noise-exposed rats compared to controls, but recover to levels similar to controls after 8 weeks in a normal auditory environment. This showed that BDNF decreases in the re-opening of the critical period by constant noise exposure, and increases during the experience-dependent plasticity that follows the critical period [13]. Interestingly patients with schizophrenia showed an increase in serum BDNF levels after performing intensive neuroplasticity-based cognitive training [26]. Here we observed that BDNF infusion increased the experience-dependent plasticity following exposure to 7 kHz, indicating that BDNF plays an active role on those processes in the auditory cortex. We started to manipulate BDNF signaling two days prior to the onset of the critical period, thus one cannot exclude that the early BDNF infusion is opening the critical period window earlier amplifying the experience-dependent plasticity induced by pure tone exposure during the critical period. Conversely we cannot exclude the possibility that the antibody against BDNF delayed the onset of the critical period, contributing to the observed effects we reported here.

We confirmed the successful modulation of BDNF signaling by reporting biochemical changes in A1 consistent with the endogenous role of BDNF. It is known that the expression of the GABA_A_ β2/3 subunit of GABA receptors is controlled by BDNF signaling [27]. In our study, the antibody against BDNF reduced the expression of the GABA_A_ β2/3 subunit in the A1. The decrease in GABA_A_ receptors may account for the immature tonotopy and the poor selectivity to tone frequencies observed in the anti-BDNF group. In the visual cortex, BDNF controls the opening and closure of critical period by modulating GABA signaling [1], [28]. In the barrel cortex, BDNF also seems to be involved in the induction of experience-dependent plasticity and in the opening of critical period, through the induction of the development of GABAergic parvalbumin-positive neurons [29].

One limitation of our study is that we were not able to determine if alterations induced by BDNF modulation are reversible after the end of the exposure to either anti-BDNF or exogenous BDNF. In previous studies, our group demonstrated that the degradation of neuronal selectivity in A1 by raising rat pups in the presence of noise endures into adulthood [7], [9], [14], [30]. Moreover, Zhou and Merzenich (2007, 2009) [31], [32] demonstrated that perceptual training applied to developmentally degraded post-critical period of rats resulted in the recovery of normal representational fidelity and tonotopy. Thus we expect that the disturbances induced by BDNF observed here would endure into adulthood. We also did not analyze the effects of BDNF manipulation on experience-dependent plasticity in the auditory cortex of adult rats. Noteworthy in the visual cortex, exogenous infusion of BDNF increases experience-dependent plasticity in kittens but has no effect in adult cats [33].

Another limitation of our study is that the rate of release of BDNF and anti-BDNF molecules from the Elvax resin to the A1 tissue could not be precisely controlled. The implanted resin itself seems not to induce alterations or injury that affects the development of A1 organization, since our control maps and unit responses were indistinguishable from those observed in prior studies [8].

Several previous studies have pointed out that, during critical period, the normal development of auditory cortex is more susceptible to poor or absent environmental auditory inputs [7]. Frequently, an impaired auditory acuity develops in this phase, which is followed by the establishment of language deficits in adulthood [6]. The present study contributes to understand the role of BDNF in the experience-dependent plasticity that shapes the auditory cortex during development. The observed effects of BDNF in A1 provide perspectives for the use of alternative therapies [34] in conjunction with cognitive training to improve the performance of language, reading and speech [16], [35], [36], [37].
